# Close linkage between serum uric acid and cardiac dysfunction in patients with ischemic heart disease according to covariance structure analysis

**DOI:** 10.1038/s41598-017-02707-y

**Published:** 2017-05-30

**Authors:** Yoshiro Tanaka, Tomohisa Nagoshi, Makoto Kawai, Goki Uno, Satoshi Ito, Akira Yoshii, Haruka Kimura, Yasunori Inoue, Kazuo Ogawa, Toshikazu D. Tanaka, Kosuke Minai, Takayuki Ogawa, Michihiro Yoshimura

**Affiliations:** Division of Cardiology, Department of Internal Medicine, The Jikei University School of Medicine, 3-25-8, Nishi-Shinbashi, Minato-ku, Tokyo, 105-8461 Japan

## Abstract

High serum uric acid (UA) level has been assumed to be a risk factor for left ventricular (LV) dysfunction; however, the precise relationship between these conditions has not been fully examined because many confounding factors are associated with UA level. We herein examined the precise relationship by proposing structural equation models. The study population consisted of 1432 cases with ischemic heart disease who underwent cardiac catheterization. Multiple regression analyses and covariance structure analyses were performed to elucidate the cause-and-effect relationship between UA level and LV ejection fraction (LVEF). A path model exploring the factors contributing to LVEF showed that high UA was a significant cause of reduced LVEF (P = 0.004), independent of other significant factors. The degree of atherosclerosis, as estimated by the number of diseased coronary vessels, was significantly affected by high UA (P = 0.005); and the number of diseased coronary vessels subsequently led to reduced LVEF (P < 0.001). Another path model exploring the factors contributing to UA level showed that LVEF was a significant cause of high UA (P = 0.001), while other risk factors were also independent contributing factors. This study clearly demonstrated that there was a close link between high UA and LV dysfunction, which was represented by possible cause-and-effect relationship.

## Introduction

Patients with high uric acid (UA) levels are predisposed to gout as a major clinical complication. With the increased awareness of the hazardousness of high UA levels, discussion has also recently resurfaced in the cardiovascular field^1–3^. According to epidemiological research and meta-analyses, the serum UA level predicts the progression of chronic kidney disease and the development of stroke^4, 5^. Furthermore, elevated UA levels are associated with the presence of hypertension, diabetes, and metabolic syndrome^6–8^, while the relationship between ischemic heart disease (IHD) and serum UA level remains controversial. Another recent meta-analysis studying the relationship between serum UA and IHD showed that a high UA level was not likely to be a main determinant of IHD and that it may not significantly contribute to the prediction of IHD in the general population^9^.

In contrast, in patients with heart failure (rather than IHD), there has been increasing evidence to indicate that high UA levels predict an increase in morbidity and mortality^2, 10^. Nonetheless, few studies have shown a precise relationship between high UA levels and cardiac function, as measured in detail by echocardiography, cardiac catheterization, and other methods. Thus, the direct relationship between cardiac systolic dysfunction and high UA levels remains unclear and an investigation should be performed in which the effects of high UA levels on the process of coronary atherosclerosis are considered.

When taken together, although it has almost been accepted that high UA levels are associated with most cardiovascular diseases, including heart failure, many more studies are required to confirm the positioning of high UA level as a risk factor for the respective cardiovascular disorders. A statistical analysis would be helpful; however, there is a degree of intractableness associated with performing a precise analysis because serum UA level is likely to be associated with many other risk factors, including -but not limited to- male gender, obesity, dyslipidemia. All of these risk factors may –both individually and collectively- affect the progression of cardiovascular diseases. It therefore seems quite difficult to confirm the risk that a high UA level itself poses in relation to cardiovascular diseases. Advanced statistical methods will be needed in order to elucidate the precise risk of high UA levels.

In a statistical analysis, confounders are extraneous variables that affect the variables being studied—when confounders are present, the results do not reflect the actual relationships between the studied variables. Confounding variables are often defined as variables that are correlated—either positively or negatively—with both the dependent variable and the independent variable. There are several ways to eliminate confounding bias, for example, by adjustment of the independent variables. Still, a confounding bias can be difficult to control if multiple potential confounding variables are present or if the study population is of insufficient size. A covariance structure analysis plays an important role in understanding how the relationships between observed variables may be generated in many areas using hypothesized latent variables. A covariance structure analysis is useful for exploratory and explanatory factor analyses. This analysis can be performed based on the confounding bias. Another merit of using a covariance structure analysis is that the analysis can package Bayesian networks to infer cause-and-effect relationships. However, the factors that are included in these analyses should be carefully selected, and the path model based on covariance structure analysis should be proposed based on consistent concepts and the clear direction of the study. In a tangible way, we successfully proposed a path model based on a covariance structure analysis in order to explain a complex phenomenon involving possible causality^11^.

In the present study, we examined the effects of high UA levels on cardiac function and the cause-and-effect relationship between them using cardiovascular disease patients, all of whom underwent cardiac catheterization in our institution. We performed a step-by-step statistical analysis to identify the risk of high UA per se for cardiac dysfunction while taking into account the influence of high UA levels on IHD—as evaluated by organic stenosis in the coronary arteries and the effect of IHD on the cardiac function.

## Results

### The characteristics of the study patients

The clinical characteristics of the 1432 cases are shown in Table 1. The mean UA level was 6.1 ± 1.4 mg/dL and the mean left ventricular ejection fraction (LVEF) was 58.7 ± 10.5%.

**Table 1:** Clinical characteristics.

Characteristics (n = 1432)	Overall; Number (%) or Mean ± SD [Median; range]
Gender; Male/Female	1235/197 (86.2/13.8)
Age (years old)	66.5 ± 11.0
BMI (kg/m^2^)	24.7 ± 3.8
Current smoker	278 (19.4)
Family history of IHD	367 (25.6)
Hb (g/dL)	13.4 ± 1.8
Cr (mg/dL)	0.93 ± 0.53
eGFR (mL/min/1.73 m^2^)	68.6 ± 19.3
UA (mg/dL)	6.1 ± 1.4
FBS (mg/dL)	118 ± 31.5
HbA1c (%)	6.3 ± 1.0
TG (mg/dL)	127.5 ± 102.3
HDL-C (mg/dL)	51.3 ± 14.7
LDL-C (mg/dL)	97.4 ± 26.6
LDL-C/HDL-C	2.04 ± 0.78
CRP (mg/dL)	0.39 ± 1.23
BNP (pg/mL)	94.8 ± 187.0 [35.7; 3.0–2520.5]
LVEF (%)	58.7 ± 10.5
*Underlying cardiovascular disease number*	
Cardiomyopathy	32 (2.2)
Valvular disease	60 (4.2)
Atrial fibrillation	60 (4.2)
Hypertension	1112 (77.7)
Diabetes mellitus	617 (43.1)
Dyslipidemia	1135 (79.3)
Renal dysfunction*	412 (28.8)
*Medication*	
ACE inhibitors	330 (23.0)
ARBs	633 (44.2)
Beta blockers	665 (46.4)
Calcium channel blockers	879 (61.4)
Diuretics	269 (18.8)
Statins	1023 (71.4)
Non-Statin for dyslipidemia	194 (13.5)
Oral antidiabetic agents	442 (30.9)
Insulin	163 (11.4)
UA lowering agents	246 (17.2)

BMI, body mass index; IHD, ischemic heart disease; Hb, hemoglobin; Cr, Creatinine; eGFR, estimated glomerular filtration rate; UA, uric acid; FBS, fasting blood sugar; HbA1c, hemoglobin A1c; TG, triglycerides; HDL-C, high-density lipoprotein; LDL-C, low-density lipoprotein; CRP, C-reactive protein; BNP, B-type natriuretic peptide; LVEF, left ventricular ejection fraction; ACE, angiotensin-converting enzyme; and ARBs, angiotensin II type I-receptor blockers.

*Renal dysfunction = eGFR < 60 mL/min/1.73 m^2^ (excluded hemodialysis-dependent patients).

### The multiple regression analysis to determine the association between risk factors, LVEF and the number of diseased vessels

A multiple regression analysis was performed to examine the effects of risk factors on the LVEF (Supplementary Table S1). The analysis revealed that age (P < 0.001), body mass index (BMI) (P < 0.001), estimated glomerular filtration rate (eGFR) (P < 0.001), triglyceride (TG) (P = 0.002), systolic blood pressure (AOsys) (P < 0.001), hemoglobin A1c (HbA1c) (P < 0.001), smoking habit (P = 0.030), and UA (P < 0.001) were significant factors. Another multiple regression analysis was performed to examine the effects of risk factors on the number of diseased vessels (Supplementary Table S2). The analysis identified age (P = 0.015), eGFR (P = 0.017), TG (P = 0.033), AOsys (P = 0.001), HbA1c (P < 0.001), and UA (P = 0.011) were significant factors.

### The multiple regression analysis to determine the factors associated with serum UA level

A multiple regression analysis was performed to examine the effect of risk factors on the serum UA level (Supplementary Table S3). The analysis identified age (P = 0.002), gender (P < 0.001), BMI (P < 0.001), eGFR (P < 0.001), TG (P < 0.001), AOsys (P = 0.017), HbA1c (P = 0.018), and LVEF (P = 0.002) as significant factors.

### The single regression analysis to search for confounding biases among the risk factors

A single regression analysis was performed in order to search for confounding biases among the independent variables. Most of the pairs showed a significant association (Supplementary Table S4). Thus, the results of the above-mentioned multiple regression analyses lost some of their meaning.

### The concept of the proposed path model (A): A high UA level as a possible cause of LV dysfunction

**Figure 1:**
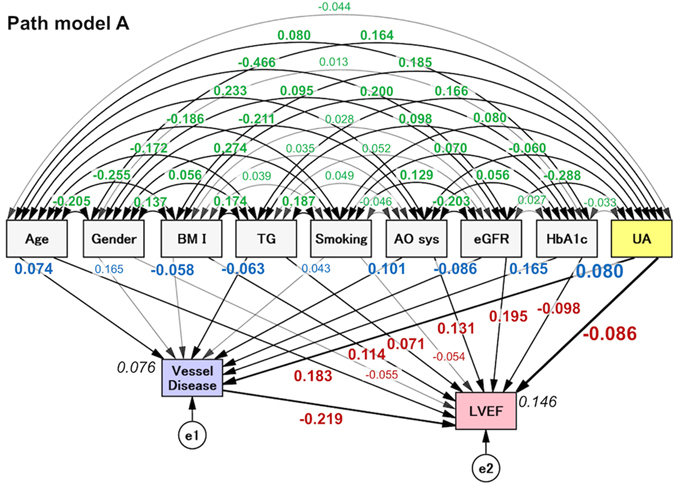
The path model [A]: An explanatory drawing of the possible cascade from risk factors to the number of diseased vessels and LVEF. This path has a coefficient showing the standardized coefficient of a regressing independent variable on a dependent variable of the relevant path. These variables indicate standardized regression coefficients (direct effect) [bold typeface indicates remarkable values], squared multiple correlations [narrow italics] and correlations among exogenous variables [green]. BMI, body mass index; TG, triglyceride; AOsys, systolic blood pressure; eGFR, estimated glomerular filtration rate; HbA1c, hemoglobin A1c; UA, uric acid; e, extraneous variable.

As a matter of logic, the theoretical path model (A) was proposed by positioning the serum UA level in parallel with the other risk factors of age, gender, BMI, TG, smoking, AOsys, eGFR, and HbA1c (Fig. 1). In order to examine possible causality, paths between variables were drawn from independent variables to dependent variables with a directional arrow for each regression model. All of the risk factors had the potential to confound each other. The association between two factors was linked by two-way arrows. In this path model, the number of vessel diseases was positioned to convert a path from all of the risk factors to LVEF because the degree of coronary artery disease is possibly influenced by all risk factors and then subsequently affects LVEF.

### The results of the path model (A)

The precise results of the path model (A) are shown in Table 2 and Supplementary Table S5. The exploratory factor analysis revealed that age, BMI, TG, AOsys, eGFR, HbA1c, and UA were significant causes of reduced LVEF. The negative correlation between UA level and LVEF was found with a significant impact (standardized regression coefficients, β: −0.086, P = 0.004). Furthermore, the exploratory factor analysis revealed that age, BMI, TG, AOsys, eGFR, HbA1c, and UA were significant causes of the number of diseased vessels. The number of diseased vessels was causally linked to a reduction of LVEF.

**Table 2:** The results of path model A.

Clinical Factor			Estimate	Standard error	Test statistic	*P*-Value	Standard regression coefficient		
							Direct Effect	Indirect Effect	Total Effect
Vesssel Disease (R^2^ = 0.076)	←	Age	0.006	0.003	2.342	*0.019*	0.074	0	0.074
	←	Gender	0.088	0.077	1.153	*0.249*	0.165	0	0.165
	←	BMI	−0.014	0.007	−2.057	*0.040*	−0.058	0	−0.058
	←	TG	−0.001	0.000	−2.319	*0.020*	−0.063	0	−0.063
	←	Smoking	0.058	0.037	1.573	*0.116*	0.043	0	0.043
	←	AO sys	0.004	0.001	3.502	<*0.001*	0.101	0	0.101
	←	eGFR	−0.004	0.002	−2.725	*0.006*	−0.086	0	−0.086
	←	HbA1c	0.161	0.026	6.142	<*0.001*	0.165	0	0.165
	←	UA	0.055	0.020	2.792	*0.005*	0.080	0	0.080
LVEF (R^2^ = 0.146)	←	Age	0.176	0.032	5.500	<*0.001*	0.183	−0.016	0.166
	←	Gender	0.088	0.885	−1.887	*0.059*	−0.055	−0.007	−0.062
	←	BMI	−0.014	0.080	3.887	<*0.001*	0.114	0.013	0.127
	←	TG	−0.001	0.003	2.502	*0.012*	0.071	0.014	0.085
	←	Smoking	−0.810	0.429	−1.889	*0.059*	−0.054	−0.009	−0.063
	←	AO sys	0.057	0.012	4.548	*<0.001*	0.131	−0.022	0.109
	←	eGFR	0.107	0.018	5.935	<*0.001*	0.195	0.019	0.214
	←	HbA1c	−1.056	0.305	−3.458	<*0.001*	−0.098	−0.036	−0.134
	←	UA	−0.652	0.227	−2.868	*0.004*	−0.086	−0.018	−0.103
	←	Vessel Disease	−2.417	0.306	−7.797	<*0.001*	−0.219.	0	−0.219

The results (direct, indirect, and total effects) of the path model theoretically proposed analysis to identify the clinical factors influencing between each other (see Fig. 1). RMSEA 0.128, AIC 154.0.

R^2^: squared multiple correlations.

BMI, body mass index; TG, triglycerides; AO sys, Systolic blood pressure in the Aorta; eGFR, estimated glomerular filtration rate; HbA1c, hemoglobin A1c; UA, uric acid; LVEF, left ventricular ejection fraction.

### The concept of proposed path models (B) and (C); LV dysfunction as a possible cause of high UA level

**Figure 2:**
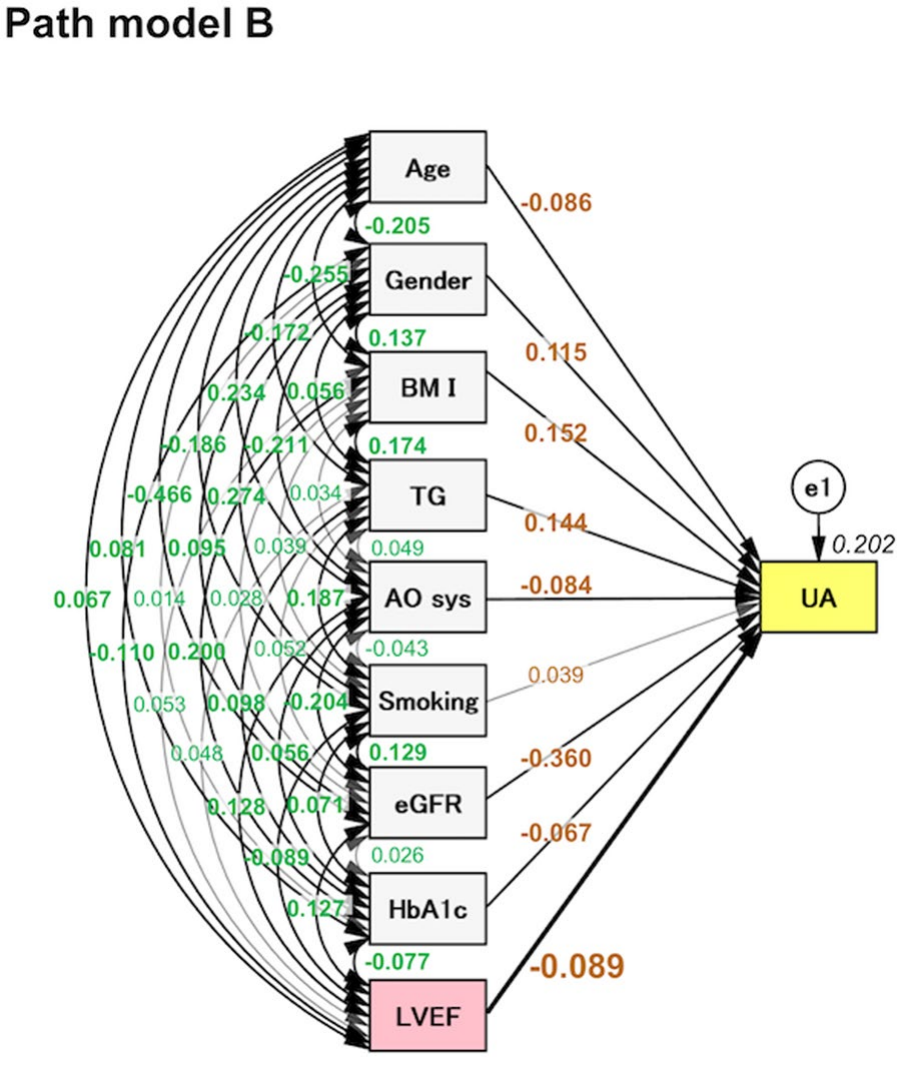
The path model [B]. An explanatory drawing of the possible cascade from risk factors to UA. Each path has a coefficient showing the standardized coefficient of a regressing independent variable on a dependent variable of the relevant path. These variables indicate standardized regression coefficients (direct effect) [bold typeface indicates remarkable values], squared multiple correlations [narrow italics] and correlations among exogenous variables [green].

The theoretical path model (B) was proposed by positioning LVEF in parallel with the risk factors of age, gender, BMI, TG, AOsys, smoking, eGFR, and HbA1c, all of which possibly affect the serum UA level (Fig. 2). The serum UA level may also be affected by the medication profile, such as the usage of UA lowering agents and diuretics. Thus, another theoretical path model (C) was proposed (Supplementary Fig. S1). All of the risk factors had the potential to confound each other. The association between two factors was linked by two-way arrows.

### The results of path models (B) and (C)

The precise results of path models (B) and (C) are shown in Tables 3 (and Supplementary Table S6) and Supplementary Table S7, respectively. The exploratory factor analysis revealed that age, gender, BMI, TG, AOsys, eGFR, HbA1c, and LVEF were significant causes of high serum UA levels (Table 3). Moreover, although the usage of diuretics were significant factors that affected the serum UA level, LVEF was consistently negatively correlated with the serum UA level independently of the medication profile (Supplementary Table S7).

**Table 3:** The results of path model B.

Clinical Factor			Estimate	Standard error	Test statistic	*P*-Value	Standard regression coefficient
							Direct Effect
UA (R^2^ = 0.202)	←	Age	−0.011	0.004	−2.843	*0.004*	−0.086
	←	Gender	0.462	0.104	4.426	<*0.001*	0.115
	←	BMI	0.055	0.009	5.811	<*0.001*	0.152
	←	TG	0.002	0.000	5.762	<*0.001*	0.144
	←	AO sys	−0.005	0.002	−3.107	*0.002*	−0.084
	←	Smoking	0.078	0.051	1.531	*0.126*	0.039
	←	eGFR	−0.026	0.002	−12.687	<*0.001*	−0.360
	←	HbA1c	−0.095	0.036	−2.669	*0.008*	−0.067
	←	LVEF	−0.012	0.004	−3.283	*0.001*	−0.089

The results (direct effect) of the path model theoretically proposed analysis to identify the clinical factors influencing between each other (see Fig. 2). RMSEA 0.133, AIC 130.0.

R^2^: squared multiple correlations.

UA, uric acid; BMI, body mass index; TG, triglycerides; AO sys, Systolic blood pressure in the Aorta; eGFR, estimated glomerular filtration rate; HbA1c, hemoglobin A1c; LVEF, left ventricular ejection fraction.

## Discussion

It has recently been reported that a high UA level is a significant risk factor for many cardiovascular diseases, including heart failure^1, 2^; however, the precise positioning of UA in these conditions remains unclear and controversial behind the high impact of metabolic syndrome. One of the difficulties in research the impact of UA is probably attributable to the intractableness of the statistical approach because the serum UA levels are tightly associated with numerous other risk factors. In this study, we used a covariance structure analysis together with single and multiple regression analyses. A covariance structure analysis can eliminate confounding bias and clarify possible cause-and-effect relationships. As a result, we found that a high UA level was causally related to LV dysfunction and *vice versa*, suggesting that there is a possible cause-and-effect linkage between these factors.

The current study showed that high UA levels were thought to be causally reduced LVEF. In this study, we took account of the severity of IHD because IHD is affected by numerous risk factors, and subsequently affects LV dysfunction. The path model (A) was proposed in order to simultaneously estimate the effects of risk factors for both IHD and LVEF in a single equation model. This analysis indicated that high UA levels were causally linked to the severity of IHD and then that IHD affected LVEF. Importantly, it was clarified that high UA levels reduced LVEF independently of the severity of IHD.

We found that an older age, male gender, obesity, hypertriglyceridemia, hypertension, renal dysfunction, and diabetic condition were major factors associated with high UA levels in Path model B. The current results are mostly in agreement with those of previous reports; hyperuricemia is closely associated with visceral fat accumulation^12, 13^ and various metabolic disorders, such as glucose intolerance, elevated blood pressure, dyslipidemia, and atherosclerotic cardiovascular diseases, which are conceptualized as metabolic syndrome^14–18^. However, smoking fell short of being significantly associated with hyperuricemia, in contrast to the findings in previous reports^19, 20^.

In this study, 85.5% of the patients were male and 14.7% were female. Generally, the serum UA level is elevated during puberty in men and after menopause in women. In addition, the UA clearance in the kidney is low in men. Therefore, there is a gender difference in the serum UA level, which might have influenced the results of the current study. However, given the small number of female patients in the study, we are unable to make a conclusive decision at present, and analyses concerning gender differences in serum UA levels should be performed in the future.

Although the molecular mechanisms underlying the harmful effects of high UA levels have not been fully examined, the activation of redox-dependent effects, extracellular regulated kinase (ERK), proinflammatory pathway, and endothelin-1^1, 3, 21, 22^ are likely involved. Furthermore, elevated UA levels reflect upregulated xanthine oxidase (XO) activity, which induces oxidative stress^2, 23, 24^. These findings suggest that a high UA level is a major risk factor for the development of cardiovascular diseases. On the other hand, this study also indicated that LV dysfunction causally induced high UA levels. The molecular mechanisms are also unclear at present; however, the proposed mechanisms are as follows: adenosine triphosphate (ATP), which is synthesized in the mitochondria is transferred to myofibril by phosphocreatine (PCr) through the Cr kinase energy shuttle, and is used by the contractile mechanism to form adenosine diphosphate (ADP), which is resynthesized by oxidative phosphorylation in the mitochondria^25–27^. In states of ATP depletion and reduced Cr kinase shuttle, such as heart failure^28^, the metabolic turnover of the purine metabolism is increased, and the breakdown paths below ADP are activated, eventually reaching the breakdown endpoint of UA by the activation of XO^27, 29^. At present, it is unclear whether this biological reaction occurs in the cardiomyocytes. Future research should be focused on the precise energetic metabolic changes that occur in connection with high UA levels in an *in vitro* analysis.

When taken together, this study suggests that high UA levels lead to the development of heart failure; and *vice versa* (namely, that heart failure induces high UA levels), drifting into a vicious cycle. The current study strongly supports the previous reports that show the significance of high UA levels as a harmful effect in heart failure^30, 31^. Overall, the present findings indicate that UA is not only an active player but also an indirect marker of the pathogenesis of IHD. XO inhibitors could theoretically reverse the harmful reactions of XO and those of high UA^32–35^. Inhibition of XO can be expected to decrease the formation of cytosolic ROS, which ought to be increased in heart failure. XO inhibitors would thus in turn lessen the inhibition imposed by ROS on the cytosolic CK, which simulates the formation of ATP from PCr, thereby providing energy for contraction in HF. In fact, a recent study reported that XO inhibitors accelerated Cr kinase energy shuttle^29^. In addition, XO inhibitors theoretically block the purine metabolism, namely, the paths of ATP breakdown. Taken together, XO inhibitors may have the potential to produce and conserve ATP in the failing heart. In this context, it would be interesting to examine the effect of XO inhibitors on LVEF independently of serum UA. Unfortunately, the number of patients taking XO inhibitors in the present study was not sufficient for a statistical analysis, and details regarding the medications being taken by patients were missing. However, the findings of such an analysis along with those of a covariance structure analysis would thus support the need to conduct a prospective tracking study of XO inhibitors in patients with heart failure.

In the present study, the mean UA level was 6.1 ± 1.4 mg/dL, and UA-lowering agents were administered to 17.2% of cases. In other words, the UA level in most subjects was considered to be within the normal range, and the number of the patients with hyperuricemia were small in this study. Given these clinical characteristics, the present results may indicate a harmful effect of not-very-high levels of serum UA on the cardiac function, although we have no way of determining the ideal levels of serum UA.

The present study is associated with some limitations. First, we used the TG level to represent dyslipidemia because we preliminarily performed an association study of UA with TG and low-density lipoprotein (LDL) and found a substantial link between TG and UA rather than LDL and UA (data not shown). In the future, we should perform an association study to determine the associations between UA and precise lipid profiles, including LDL, HDL, TG, and oxidized-LDL using a covariance structure analysis and attempt to clarify the combinations that are truly hazardous with regard to the progression of coronary atherosclerosis and heart failure. Second, the mean age of the subjects was 66.5 years old, which seems relatively young among subjects with IHD. This population characteristic is believed to be due to the exclusion of elderly or critically ill patients who did not meet the indications for cardiac catheterization. There may therefore be a selection bias among our study patients, and further studies in a more general population are warranted. It will also be interesting to investigate the pathological conditions of IHD in greater detail, such as by dividing patients into groups of acute coronary syndrome (excluded in the current study) and stable effort angina. Furthermore, patients with non-IHD were excluded from this study; a study of non-IHD patients should be performed in another series. Third, atrial fibrillation is likely to have an important association with high UA levels^36, 37^. However, this study did not include precise data about AF. This important analysis should also be performed in the future. Finally, it is quite natural for serum levels of UA to change substantially over time and due to the effects of various drugs, although diuretics are typically stopped before catheterization in our facility. Food intake, alcohol consumption and physical stress also contribute to rapid changes in the serum UA. In contrast, the progress of atherosclerosis and the development of heart failure is relatively slow in general. However, we used the serum UA level obtained at the point of cardiac catheterization in this study. Although the study population was relatively large, this represents another study limitation.

In summary, while elevated UA levels are often associated with established cardiovascular risk factors, the question as to whether UA is an independent risk factor for IHD and/or heart failure remains controversial. This is probably attributable to the intractableness of statistical approaches because the serum UA levels are tightly associated with many other risk factors. In the present study, we examined the relative importance of the associations between UA levels and cardiac function, with consideration for the severity of IHD using a covariance structure analysis. After eliminating multiple confounding biases, we found that a high UA level is thought to be causally related to LV dysfunction and *vice versa*, indicating that there is a direct cause-and-effect linkage between these factors. Stratified analyses, for example, by gender difference and the degree of obesity, are needed to further explore the condition in which this linkage becomes more evident.

## Methods

### Study patients

The study population consisted of 1432 cases with IHD who were consecutively admitted to our institutions from 2012 to 2016. All of the patients underwent cardiac catheterization for an evaluation of IHD. We excluded the patients who underwent hemodialysis because their cardiac function was significantly altered by artificial volume control. Emergency cases (i.e. acute coronary syndrome) were also excluded, as their hemodynamics (including LVEF) and miscellaneous biomarkers are highly variable and do not reflect the values under stable conditions. The ethics committee of The Jikei University School of Medicine approved the study protocol (24–355[7121]), and we complied with the routine ethical regulations of our institution. This was a retrospective study, and informed consent could not be obtained from each patient. Instead of obtaining informed consent from each patient, we posted a notice about the study design and contact information at a public location in our institution.

### The disease definitions

IHD was diagnosed based on symptoms, electrocardiography (ECG), blood sampling, and the coronary artery morphology. Organic lesions producing ≥75% luminal stenosis of the coronary arteries on coronary angiography were defined based on the modified American Heart Association (AHA) coronary tree segment classification^38^. The number of diseased vessels was counted as the number of three major coronary arteries (i.e. left anterior descending [LAD], left circumflex [LCx] and right coronary arteries) with organic lesions that were indicated for treatment by revascularization and/or standard medical therapy. Diagonal and high lateral branches were included in LAD and LCx, respectively, if they had a substantial myocardial perfusion area and were indicated for treatment. In the present study, we divided the patients into four groups based on the number of diseased vessels with significant organic stenosis (the 0-, 1-, 2-, and 3-vessel groups). Patients with a left main trunk lesion were included in the 2-vessel group. The number of diseased vessels was counted at the time of cardiac catheterization in this study, and lesions already treated by revascularization were not included. Patients with coronary spasm (diagnosed on the basis of clinical findings, including ECG change, or a provocation test with intracoronary injection of acetylcholine) were placed into the 0-vessel group if there was no organic stenosis after nitroglycerin administration. Some of the patients had comorbid cardiovascular diseases, such as valvular disease, arrhythmia, cardiomyopathy, and other conditions. Hypertension, Diabetes mellitus (DM) and dyslipidemia were defined as described previously^39^. The eGFR was calculated according to the Modification of Diet in Renal Disease (MDRD) Study equation shown below^40^, with coefficients modified for Japanese patients^41^.

$${\rm{eGFR}}({\rm{mL}}/\,{\rm{\min }}\,/\,1.73{{\rm{m}}}^{2})=194\times {{\rm{age}}}^{-0.287}\times {{\rm{Creatinine}}}^{-1.094}\,({\rm{and}}\times 0.739\,{\rm{for}}\,{\rm{females}}).$$

Renal dysfunction was defined by an eGFR of <60 mL/min/1.73 m^2^, according to the guidelines of the Japanese Society of Nephrology.

### Blood sampling and hemodynamic examinations during cardiac catheterization

We collected blood samples and hemodynamic data during cardiac catheterization. The serum biochemical analyses were performed in a central laboratory of our hospital during the study period. In this statistical analysis, we included serum levels of Cr, UA, and TG, AOsys, and HbA1c level. We included smoking habit (0 for non-smoker, 1 for past smoker, and 2 for current smoker). LVEF was measured at the time of left ventriculography^42^.

### Statistical analysis

Continuous variables are expressed as the mean ± standard deviation (SD) or medians. The correlation between two factors was investigated by a single regression analysis and expressed as Spearman’s correlation coefficient. A multiple regression analysis was performed to compare multiple values. The above-mentioned statistical analyses were performed using the SPSS Statistics software program (version 23.0, SPSS Inc., Chicago, IL, USA). P values of <0.05 were considered to indicate statistical significance.

A path analysis based on a covariance structure analysis was used to investigate the relationship between clinical factors in this study population and to survey the probable causal effects on LVEF or serum UA level. The path analysis was performed using the IBM SPSS AMOS software program (version 23, Amos Development Corporation, Meadville, PA, USA). We have previously described how to write a path model^11^. In brief, the possible causality model defined some hierarchical regression models between clinical factors and LVEF or serum UA level. For every regression, the total variance in dependent variable is theorized to be caused by either independent variables that are included in the model or by extraneous variables (e). The indirect effect was determined by multiplying the path coefficients of the intervening variables. The structural equation models that were obtained were tested and confirmed; P values of < 0.05 were considered to indicate statistical significance.

## Electronic supplementary material

Supplementary Information
