# Supplementary material for: Close linkage between serum uric acid and cardiac dysfunction in patients with ischemic heart disease according to covariance structure analysis
Source: Sci Rep. 2017 May 30;7:2519. doi: 10.1038/s41598-017-02707-y (PMC5449391; doi:10.1038/s41598-017-02707-y)
Supplement: Supplementary file 1 — Supplementary Information [file 41598_2017_2707_MOESM1_ESM.pdf]

Supplementary Information

Close linkage between serum uric acid and cardiac dysfunction in patients with ischemic heart disease according to covariance structure analysis

Yoshiro Tanaka, Tomohisa Nagoshi, Makoto Kawai, Goki Uno, Satoshi Ito, Akira Yoshii, Haruka Kimura, Yasunori Inoue, Kazuo Ogawa, Toshikazu D. Tanaka, Kosuke Minai Takayuki Ogawa, Michihiro Yoshimura

Supplementary Table S1. The results of the multiple regression analysis to identify the clinical factors influencing the LVEF value

R^2=0.083	Non-Standard Coefficient		Standard Regression Coefficient	Test statistic	*P-value*	95% CI	VIF
	Regression Coefficient	Standard Error					
Age	0.157	0.034	0.161	4.589	*< 0.001*	0.090 to 0.224	1.555
Gender	-1.526	0.920	-0.051	-1.658	*0.098*	-3.331 to 0.280	1.190
BMI	0.317	0.085	0.117	3.738	*< 0.001*	0.151 to 0.483	1.238
eGFR	0.113	0.021	0.182	5.438	*< 0.001*	0.072 to 0.154	1.421
UA	-0.762	0.243	-0.098	-3.137	*0.002*	-1.238 to -0.285	1.232
TG	0.009	0.003	0.091	3.083	*0.002*	0.003 to 0.014	1.117
AO sys	0.046	0.013	0.106	3.574	*< 0.001*	0.021 to 0.071	1.110
HbA1c	-1.399	0.309	-0.132	-4.526	*< 0.001*	-2006 to -0.793	1.085
Smoking	-0.970	0.446	-0.066	-2.175	*0.030*	-1.845 to -0.095	1.159
constant	40.957	4.863	-	8.422	*< 0.001*	31.416 to 50.499	-

R^2: adjusted coefficient of determination, CI: confidence interval, VIF: variance inflation factor.

LVEF, left ventricular ejection fraction; BMI, body mass index; eGFR, estimated glomerular filtration rate; UA, uric acid; TG, triglycerides; AO sys, Systolic blood pressure in the Aorta; HbA1c, hemoglobin A1c

Supplementary Table S2. The results of the multiple regression analysis to identify the clinical factors influencing with or without the vessel disease

R^2=0.070	Non-Standard Coefficient		Standard Regression Coefficient	Test statistic	*P-value*	95% CI	VIF
	Regression Coefficient	Standard Error					
Age	0.008	0.003	0.085	2.446	*0.015*	0.002 to 0.014	1.600
Gender	0.071	0.084	0.025	0.841	*0.400*	-0.094 to 0.235	1.186
BMI	-0.015	0.008	-0.059	-1.912	*0.056*	-0.030 to 0.000	1.239
eGFR	-0.004	0.002	-0.080	-2.381	*0.017*	-0.008 to -0.001	1.509
UA	0.054	0.021	0.078	2.536	*0.011*	0.012 to 0.096	1.247
TG	-0.001	0.000	-0.062	-2.137	*0.033*	-0.001 to 0.000	1.112
AO sys	0.004	0.001	0.099	3.374	*0.001*	0.002 to 0.006	1.132
HbA1c	0.159	0.028	0.161	5.609	< *0.001*	0.104 to 0.215	1.090
Smoking	0.067	0.041	0.048	1.637	*0.102*	-0.013 to 0.147	1.157
constant	-0.924	0.441	-	-2.098	*0.036*	-1.789 to -0.060	-

R^2: adjusted coefficient of determination, CI: confidence interval, VIF: variance inflation factor.

BMI, body mass index; eGFR, estimated glomerular filtration rate; UA, uric acid; TG, triglycerides; AO sys, Systolic blood pressure in the Aorta; HbA1c, hemoglobin A1c

Supplementary Table S3. The results of the multiple regression analysis to identify the clinical factors influencing the UA level

R^2=0.189	Non-Standard Coefficient		Standard Regression Coefficient	Test statistic	*P-value*	95% CI	VIF
	Regression Coefficient	Standard Error					
Age	-0.013	0.004	-0.105	-3.161	*0.002*	-0.021 to -0.005	1.570
Gender	0.543	0.110	0.141	4.935	< *0.001*	0.327 to 0.758	1.168
BMI	0.053	0.010	0.153	5.235	< *0.001*	0.033 to 0.073	1.224
eGFR	-0.026	0.002	-0.322	-10.565	< *0.001*	-0.030 to -0.021	1.329
TG	0.002	0.000	0.150	5.406	< *0.001*	0.001 to 0.002	1.099
AO sys	-0.004	0.002	-0.067	-2.395	*0.017*	-0.007 to -0.001	1.117
HbA1c	-0.089	0.038	-0.066	-2.372	*0.018*	-0.163 to -0.015	1.099
Smoking	0.088	0.054	0.046	1.628	*0.104*	-0.018 to 0.193	1.161
LVEF	-0.011	0.004	-0.086	-3.137	*0.002*	-0.018 to -0.004	1.090
constant	8.274	0.553	-	14.958	< *0.001*	7.189 to 9.360	-

R^2: adjusted coefficient of determination, CI: confidence interval, VIF: variance inflation factor.

UA, uric acid; BMI, body mass index; eGFR, estimated glomerular filtration rate; TG, triglycerides; AO sys, Systolic blood pressure in the Aorta; HbA1c, hemoglobin A1c; LVEF, left ventricular ejection fraction

Supplementary Table S4. The results of Pearson's product-moment correlation coefficient analysis among the clinical factors

	Age	Gender	BMI	TG	Smoking	AO sys	eGFR	HbA1c	UA
Age	-	-0.205^{**}	-0.255^{**}	-0.174^{**}	-0.186^{**}	0.223^{**}	-0.462^{**}	0.075^{**}	-0.044
Gender	-0.205^{**}	-	0.137^{**}	0.055^{**}	0.274^{**}	-0.208^{**}	0.091^{**}	0.016	0.165^{**}
BMI	-0.255^{**}	0.137^{**}	-	0.175^{**}	0.039	0.025	0.032	0.199^{**}	0.185^{**}
TG	-0.174^{**}	0.055^{*}	0.175^{**}	-	0.187^{**}	0.050	0.052	0.098^{**}	0.166^{**}
Smoking	-0.186^{**}	0.274^{**}	0.039	0.187^{**}	-	-0.048	0.131^{**}	0.070^{**}	0.079^{**}
AO sys	0.223^{**}	-0.208^{**}	0.025	0.050	-0.048	-	-0.194^{**}	0.053	-0.064^{*}
eGFR	-0.462^{**}	0.091^{**}	0.032	0.052	0.131^{**}	-0.194^{**}	-	0.029	-0.287^{**}
HbA1c	0.075^{**}	0.016	0.199^{**}	0.098^{**}	0.070^{**}	0.053	0.029	-	-0.034
UA	-0.044	0.165^{**}	0.185^{**}	0.166^{**}	0.079^{**}	-0.064^{*}	-0.287^{**}	-0.034	-

BMI, body mass index; TG, triglycerides; AO sys, Systolic blood pressure in the Aorta; eGFR, estimated glomerular filtration rate; HbA1c, hemoglobin A1c; UA, uric acid

P value: $^{*}P < 0.05$, $^{**}P < 0.01$

Supplementary Table S5. Correlation coefficients of each clinical factor from the results of path model A

Age	↔	Gender	-0.205**
	↔	BMI	-0.255**
	↔	TG	-0.172**
	↔	Smoking	-0.186**
	↔	AO sys	0.233**
	↔	eGFR	-0.466**
	↔	HbA1c	0.080**
	↔	UA	-0.044
Gender	↔	BMI	0.137**
	↔	TG	0.056*
	↔	Smoking	0.274**
	↔	AO sys	-0.211**
	↔	eGFR	0.095**
	↔	HbA1c	0.013
	↔	UA	0.164**
BMI	↔	TG	0.174**
	↔	Smoking	0.039
	↔	AO sys	0.035
	↔	eGFR	0.028
	↔	HbA1c	0.200**
	↔	UA	0.185**
TG	↔	Smoking	0.187**
	↔	AO sys	0.049
	↔	eGFR	0.052
	↔	HbA1c	0.098**

	↔	UA	0.166**
Smoking	↔	AO sys	-0.046
	↔	eGFR	0.129**
	↔	HbA1c	0.070**
	↔	UA	0.080**
AO sys	↔	eGFR	-0.203**
	↔	HbA1c	0.056*
	↔	UA	-0.060*
eGFR	↔	HbA1c	0.027
	↔	UA	-0.288**
HbA1c	↔	UA	-0.033

P value: *$P < 0.05$, **$P < 0.01$

BMI, body mass index; TG, triglycerides; AO sys, Systolic blood pressure in the Aorta; eGFR, estimated glomerular filtration rate; HbA1c, hemoglobin A1c; UA, uric acid; LVEF, left ventricular ejection fraction

Supplementary Table S6. Correlation coefficients of each clinical factor from the results of path model B

Age	↔	Gender	-0.205**
	↔	BMI	-0.255**
	↔	TG	-0.172**
	↔	AO sys	0.234**
	↔	Smoking	-0.186**
	↔	eGFR	-0.466**
	↔	HbA1c	0.081**
	↔	LVEF	0.067*
Gender	↔	BMI	0.137**
	↔	TG	0.056*
	↔	AO sys	-0.211**
	↔	Smoking	0.274**
	↔	eGFR	0.095**
	↔	HbA1c	0.014
	↔	LVEF	-0.110**
BMI	↔	TG	0.174**
	↔	AO sys	0.034
	↔	Smoking	0.039
	↔	eGFR	0.028
	↔	HbA1c	0.200**
	↔	LVEF	0.053
TG	↔	AO sys	0.049
	↔	Smoking	0.187**
	↔	eGFR	0.052

	↔	HbA1c	0.098**
	↔	LVEF	0.048
AO sys	↔	Smoking	-0.043
	↔	eGFR	-0.204**
	↔	HbA1c	0.056*
	↔	LVEF	0.128**
Smoking	↔	eGFR	0.129**
	↔	HbA1c	0.071**
	↔	LVEF	-0.089**
eGFR	↔	HbA1c	0.026
	↔	LVEF	0.127**
HbA1c	↔	LVEF	-0.077**

P value: $^{*}P < 0.05$, $^{**}P < 0.01$

UA, uric acid; BMI, body mass index; TG, triglycerides; AO sys, Systolic blood pressure in the Aorta; eGFR, estimated glomerular filtration rate; HbA1c, hemoglobin A1c; LVEF, left ventricular ejection fraction

Supplementary Table S7. The results of path model C

Clinical Factor			Estimate	Standard error	Test statistic	*P*-Value	Standard regression coefficient<br>Direct Effect
UA (R^2=0.046)	←	UA lowering agents	0.127	0.098	1.297	*0.195*	0.035
	←	Diuretics	0.521	0.099	5.291	< *0.001*	0.147
	←	LVEF	-0.014	0.004	-3.648	< *0.001*	-0.108

The results (direct effect) of the path model theoretically proposed analysis to identify the clinical factors influencing between each other (see Supplementary Fig.S1)

R^2: squared multiple correlations.

Correlation coefficients of each clinical factor

UA lowering agents	↔	Diuretics	0.174**
	↔	LVEF	-0.115**
Diuretics	↔	LVEF	-0.286**

P value: $^{**}P < 0.01$

UA, uric acid; LVEF, left ventricular ejection fraction

Supplementary Figure Legend

Supplementary Figure S1. The path model [C]: An explanatory drawing of the possible cascade from the medication profiles to UA

This path has a coefficient showing the standardized coefficient of a regressing independent variable on a dependent variable of the relevant path. These variables indicate standardized regression coefficients (direct effect) [bold typeface indicates remarkable values], squared multiple correlations [narrow italics] and correlations among exogenous variables [green].

Path model C

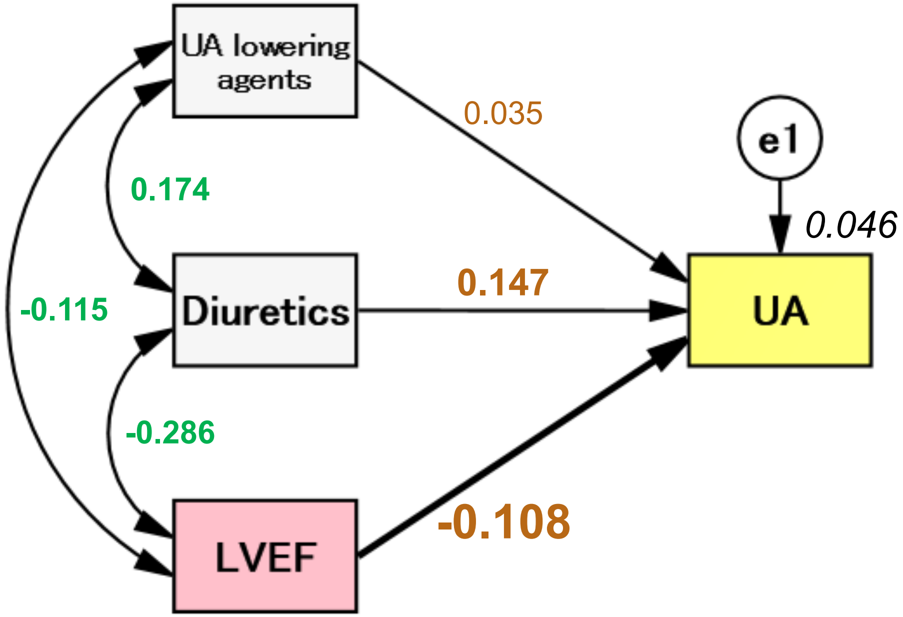

Supplementary Figure S1
